# Fungal-Derived Chitosan from *In Vitro* Mushroom Cultures as an Antimicrobial Matrix for Silver Nanoparticles in Advanced Bioactive Materials

**DOI:** 10.3390/ma18235342

**Published:** 2025-11-27

**Authors:** Agata Krakowska, Dominik Műller, Anna Kula, Iwona Skiba-Kurek, Beata Paczosa-Bator, Bożena Muszyńska, Tomasz Skalski

**Affiliations:** 1 AGH University of Krakow, Faculty of Materials Science and Ceramics, Department of Analytical Chemistry and Biochemistry, Al. Mickiewicza 30, 30-059 Kraków, Poland; domul@agh.edu.pl; 2 Department of Inorganic Chemistry and Pharmaceutical Analytics, Faculty of Pharmacy, Jagiellonian University Medical College, 9 Medyczna Street, 30-688 Kraków, Poland; 3 AGH University of Krakow, Faculty of Non-Ferrous Metals, Al. Mickiewicza 30, 30-059 Kraków, Poland; kula@agh.edu.pl; 4 Department of Microbiology, University Hospital, Marii Orwid 11 Street, 30-688 Kraków, Poland; iskiba@su.krakow.pl; 5 Department of Medical Plant and Mushroom Biotechnology, Faculty of Pharmacy, Jagiellonian University Medical College, 9 Medyczna Street, 30-688 Kraków, Poland; 6 Biotechnology Centre, Silesian University of Technology, Krzywoustego 8 Street, 44-100 Gliwice, Poland

**Keywords:** chitosan, biomass from *in vitro*, matrix, AgNPs, wound dressing

## Abstract

This study investigates chitosan extracted from *in vitro* cultures of *Hericium erinaceus* and *Pleurotus ostreatus* mushrooms as a novel antimicrobial matrix. The physicochemical properties including specific surface area, pore volume, and molecular structure were characterized by BET, SEM, and FTIR-ATR analyses. Chitosan from *P. ostreatus* exhibited a higher specific surface area (0.39 m^2^/g) compared to *H. erinaceus* (0.73 m^2^/g) and commercial chitosan (1.16 m^2^/g), correlating with enhanced antimicrobial activity against Gram-negative and Gram-positive bacterial strains. Antibacterial efficacy was quantitatively evaluated by inhibition zone diameters, with *P. ostreatus* chitosan combined with silver nanoparticles achieving an average zone of 18.2 ± 0.5 mm against *Escherichia coli*, a 25% increase compared to chitosan alone. Thermal analysis showed improved stability upon silver modification, with endothermic peak shifts from 85 °C to 118 °C. These results demonstrate that fungal-derived chitosan, particularly from *P. ostreatus*, provides a bioactive matrix with significant antibacterial properties, supporting its potential for biomedical applications. The incorporation of quantitative metrics enhances the robustness and reproducibility of the findings.

## 1. Introduction

Chitosan is a polysaccharide, specifically (1→4)-2-amine-2-deoxy-β-D-glucan. It is widely used in the medical field, primarily as a wound dressing, due to its high bioavailability, biocompatibility, and inherent antimicrobial and hemostatic properties [1,2,3,4,5]. Most commercial chitosan is currently produced from chitin extracted from marine waste, particularly crustacean shells [6,7,8]. The main advantage of this source is the large volume of waste and its limited applications outside of chitin production, allowing repurposing of material that would otherwise be discarded. However, this source has drawbacks, including dependence on fishing seasons, variability in product quality and quantity caused by the diverse diets of crustaceans, and toxic waste generated during chitin processing. An alternative source of chitin is mushroom biomass, especially *in vitro* mushroom cultures grown in controlled, specialized environments. Mushrooms contain up to 15% chitin by weight in their fungal cell walls, which is comparable to crustacean sources [9,10]. Using *in vitro* mushroom cultures eliminates seasonality and reduces variability in the final product quality.

Research on acquiring and utilizing biomass from *in vitro* cultures as a natural source of chitosan is well justified. In this study, two mushroom species were selected: Lion’s Mane Mushroom (*Hericium erinaceus*) and the popular oyster mushroom (*Pleurotus ostreatus*). Both species are widely used in traditional herbal medicine in East Asia, mainly China and Japan, due to their well-documented medicinal and health-promoting properties [11,12,13]. Beyond their antioxidant, antimicrobial, and neuroprotective effects, *H. erinaceus* and *P. ostreatus* have demonstrated anticancer activity. Their biomass has been explored in numerous studies for developing novel biomaterials for clinical applications, including the extraction of *H. erinaceus* polysaccharides (HEP) and chitin [14]. Notably, *in vitro* cultivation allows consistent and efficient production of *H. erinaceus* and *P. ostreatus* biomass to serve as an organic matrix for antibacterial applications [15]. Furthermore, antimicrobial efficacy can be enhanced by combining this biomass with metal nanoparticles, such as silver. Silver has been used for millennia due to its validated antimicrobial properties and, in nanoparticle form (AgNPs) [16], is actively applied in the pharmaceutical industry. AgNPs exhibit antimicrobial activity against a broad spectrum of pathogens, including multidrug-resistant Gram-negative and Gram-positive bacteria [17], as well as fungi. Their antimicrobial action results from both their chemical properties and nanoscale size.

Silver nanoparticles (AgNPs) exhibit natural antimicrobial properties by interacting with bacterial cell membranes [18,19]. They penetrate and disrupt membrane integrity through electrostatic interactions, causing structural damage, depolarization, and increased permeability, which leads to cellular breakdown and cell death. Silver ions released from AgNPs also damage cellular DNA and RNA, inhibiting replication, generate reactive oxygen species that disrupt cellular respiration, and interfere with electron transfer by binding to thiol groups within the cell. The antimicrobial effects of AgNPs result from both the continuous release of silver ions and their high surface energy, which compromise cell membrane integrity. Beyond infection control, AgNPs promote wound healing by modulating inflammation, stimulating fibroblast migration, and enhancing collagen deposition.

The combination of an organic matrix—fungal chitosan—with silver nanoparticles (AgNPs) presented in this study may contribute to the development of next-generation antimicrobial wound dressings, usable alongside conventional antimicrobial agents. Fungal-derived chitosan possesses a unique structure characterized by a low degree of crystallinity, which enhances its sorption capacity for substances in the wound environment, facilitating the removal of toxins from the wound bed [20]. Additionally, fungal chitosan is well tolerated by the body (characterized by low allergenic potential) due to the absence of tropomyosin, unlike crustacean-derived chitosan, making it particularly suitable for wound care applications [21]. The integration of AgNPs with chitosan matrices further enhances antimicrobial efficacy and promotes wound healing through synergistic effects, including antibacterial activity, modulation of inflammation, fibroblast migration, and collagen deposition [22,23].

This research focuses on extracting chitosan from *in vitro* cultures of *H. erinaceus* and *P. ostreatus* biomass combined with synthesized AgNPs to develop a biocompatible, effective antimicrobial matrix for advanced wound dressings. These nanocomposites have demonstrated high antimicrobial activity against common pathogens and accelerated wound healing while maintaining cytocompatibility [24,25]. The study addresses the need for consistent-quality fungal chitosan sources and optimized combinations with AgNPs to maximize clinical wound care benefits [26].

The aims of this study are to obtain and characterize a biocompatible antimicrobial matrix by extracting chitosan from the biomass of *in vitro* cultures of *H. erinaceus* and *P. ostreatus* and to combine this fungal-derived chitosan with synthesized silver nanoparticles (AgNPs). This research seeks to evaluate the antimicrobial efficacy and wound healing potential of these chitosan-AgNPs nanocomposites as advanced wound dressing materials. Specifically, the study aims to leverage the unique physicochemical properties of fungal chitosan—such as its low crystallinity and hypoallergenic nature—and the potent antibacterial, anti-inflammatory, and tissue regenerative properties of AgNPs to create synergistic materials that overcome limitations of current wound care products [27,28,29]. Through this approach, the study addresses variability issues in chitosan sources and aims to optimize the combination therapy for effective clinical application in wound management

## 2. Materials and Methods

### 2.1. The Scheme of the Experiment

**Figure 1:**
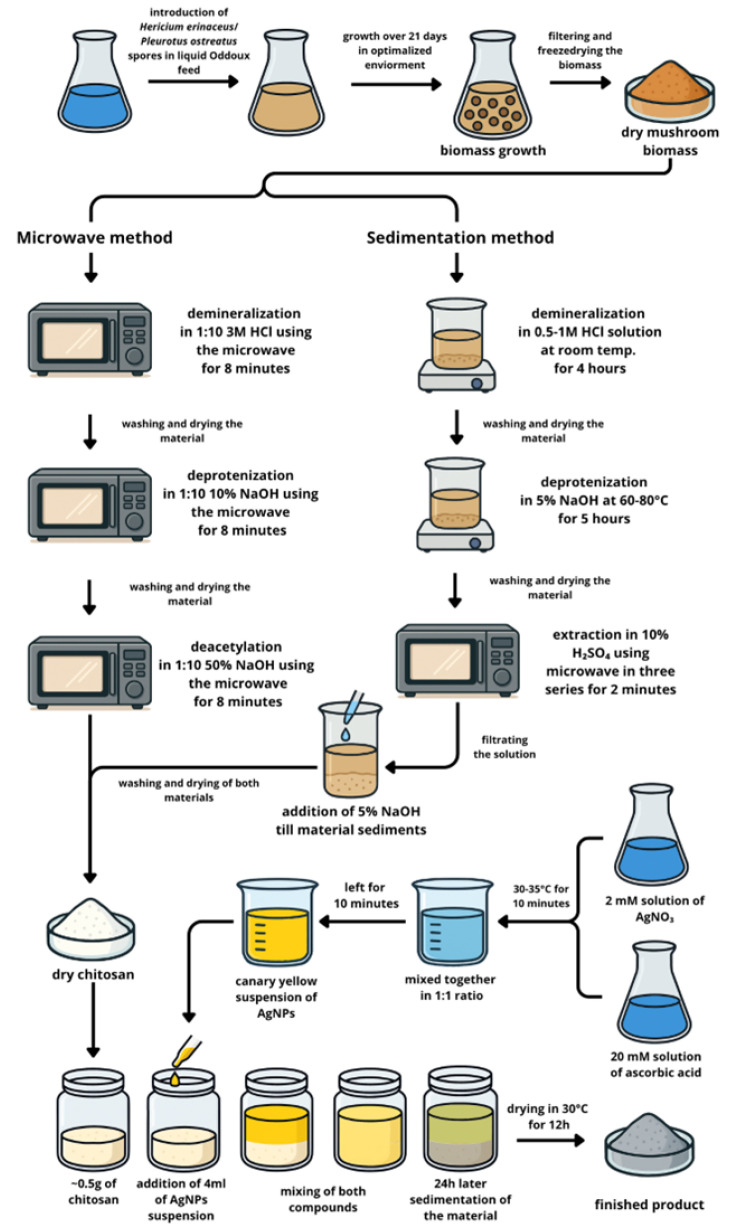
Experimental scheme illustrating the overall design and key steps of the study.

To investigate the potential use of chitosan obtained from the biomass of *in vitro* cultures of *Hericium erinaceus* and *Pleurotus ostreatus* as a matrix for silver nanoparticles, the experimental design was divided into two main stages, as illustrated in Figure 1.

**STAGE 1**—Material preparation

Step 1: Cultivation of *in vitro* mushroom mycelia from *H. erinaceus* and *P. ostreatus*

Step 2: Chitosan extraction from *in vitro* culture biomass from *H. erinaceus* and *P. ostreatus*

Step 3: Synthesis AgNPs

Step 4. Sorption of AgNPs on chitosan

**STAGE 2**—Materials analysis

The first stage involved material preparation, which included cultivating mushroom mycelia from *H. erinaceus* and *P. ostreatus in vitro*, extracting chitosan from the resulting biomass, synthesizing silver nanoparticles (AgNPs), and then adsorbing these AgNPs onto the chitosan matrix. The second stage focused on comprehensive material analysis to characterize the prepared composites and assess their properties.

### 2.2. Materials Preparation

#### 2.2.1. Biomass Material—*In Vitro* Cultures of *H. erinaceus* and *P. ostreatus* (Step 1)

In this study, three mushroom species with different ecological preferences were investigated, including *H. erinaceus* and *P. ostreatus*. To initiate the *in vitro* cultures, 0.2 g of inoculum was transferred from starter strains derived from shaken *in vitro* cultures with verified genotypes. The cultures were maintained in 500 mL Erlenmeyer flasks containing 250 mL of liquid medium prepared according to Oddoux’s formulation. Experimental conditions were set at 22 ± 2 °C and an illumination of 200 lux, with a light–dark cycle of 12–16 h light and 8–12 h dark, based on previously optimized parameters [30].

The study material consisted of the biomass of *H. erinaceus* and *P. ostreatus* grown in Oddoux liquid medium supplemented with the biometals—zincum and calcium (added as CaCl_2_ and ZnCl_2_) at a concentration of 500 mg/L, expressed as pure metal content. Cultures were prepared separately for each metal as well as for their mixture, with all variants conducted in triplicate (see Figure 1 for experimental design).

Each culture variant was performed in five independent replicates. After 21 days, corresponding to the period of optimal mycelial growth, the fungal biomass was separated from the liquid phase by filtration using a Pyrex Büchner funnel with a perforated plate (Merck, Darmstadt, Germany). Half of the obtained mycelium was used for desorption analysis, while the remaining portion was freeze-dried at –40 °C using a Labconco FreeZone 4.5 freeze dryer (Labconco, Kansas City, MO, USA). The lyophilized biomass was subsequently mineralized (each in triplicate), and the metal content was quantified using atomic absorption spectrometry (AAS). Results are expressed as mean values ± standard deviation.

#### 2.2.2. Chitosan Extraction (Step 2)

##### Chitosan Extraction from *In Vitro* Cultures of *H. erinaceus*

The first chitosan batch (C_He_—chitosan from *H. erinaceus* was prepared using a microwave-assisted extraction method. Solutions of NaOH and HCl (Merck, Darmstadt, Germany) were used in the process. For chitosan extraction, 10 g of ground biomass was initially demineralized by treatment with 3 M HCl at a 1:10 ratio in a microwave at 540 W for 8 min [31]. After thorough washing and drying, the powder was treated with 10% NaOH at a 1:10 ratio in a microwave at 150 W for 8 min to deproteinize. The resulting mass was washed, dried, and then treated with 50% NaOH at a 1:20 ratio in a microwave at 350 W for 8 min. The final chitosan product was again washed thoroughly and dried.

##### Chitosan Extraction from *In Vitro* Cultures of *P. ostreatus*

The second chitosan batch (C_Po_—chitosan from *P. ostreatus* was prepared using a precipitation method. Solutions of NaOH, HCl, and H_2_SO_4_ (all reagents from Merck, Darmstadt, Germany) were used. The raw biomass was first treated with a 0.5–1 M HCl solution at room temperature for 4 h with occasional stirring to demineralize, followed by thorough washing with distilled water. Next, the material was deproteinized using 5% NaOH solution at 60–80 °C for 5 h, then washed again with distilled water. To extract chitosan, the product underwent three microwave-assisted extractions with 10% H_2_SO_4_ solution, each lasting 2 min [32]. The pH of the solution was monitored, after which it was centrifuged and filtered. A 5% NaOH solution was then added dropwise to the filtrate to raise the pH and precipitate the chitosan. The resulting sediment was filtered, thoroughly washed with distilled water, and dried at 30 °C for several hours. For reference, a commercial Chitosan (Sigma-Aldrich, St. Louis, MO, USA, No. 448877 50G) was used.

#### 2.2.3. Synthesis of AgNPs (Steps 3 and 4)

##### Stock Solution Preparation

A 2 mM stock solution of Ag(I) was prepared by dissolving 0.0034 g of silver nitrate (AgNO_3_, Alfa Aesar, Silver nitrate, Premion, 99.995%, Merck, Darmstadt, Germany) in dimethyl sulfoxide (DMSO) using a volumetric flask. L-ascorbic acid (p.a., POCH, Gliwice, Poland) was utilized as the reducing agent. To obtain a 20 mM solution, 0.0352 g of the compound was dissolved in DMSO in a volumetric flask. Prior to mixing, both solutions containing metal precursor as well as reducing agent, were equilibrated in a water bath at 30 °C for 10 min.

##### Synthesis of Nanoparticles

**Figure 2:**
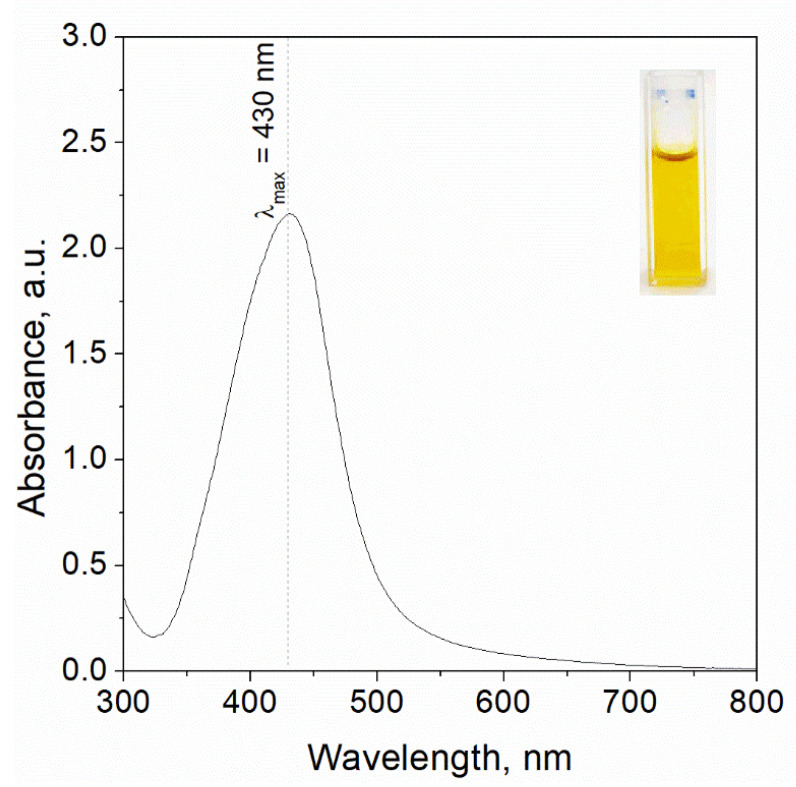
UV-Vis spectrum registered for the solution containing silver nanoparticles synthesised as a result reduction reaction of Ag(I) ions using ascorbic acid. Conditions: 2 mM Ag(I), 20 mM AA, DMSO was used as solvent, T = 20 °C. Photo of the colloidal silver (upper right corner of the chart) as well as analysis were performed after 20 min. since reagents were mixed.

Silver nanoparticles (AgNPs) were synthesized via a chemical reduction method [33] by mixing stock solutions of Ag(I) and L-ascorbic acid in a 1:1 volumetric ratio [34], according to our previous procedure. A distinct color change from colorless, attributed to unreacted precursors, to an intense yellow hue was observed, indicating nanoparticle formation [35]. The emergence of the yellow coloration, along with the presence of the Tyndall effect, confirmed the dispersion of metallic silver within the solution (see Figure 2).

### 2.3. Method Analysis

#### 2.3.1. Microstructure Analysis

The morphology of the starting materials—C_c_ (commercial chitosan derived from crustaceans), C_He_ (chitosan extracted from *H. erinaceus*), and C_Po_ (chitosan extracted from *P. ostreatus*)—as well as the chitosan composites after incorporation with silver nanoparticles, was analyzed by scanning transmission electron microscopy (STEM). A Hitachi SU-70 ultrahigh-resolution analytical Schottky emission microscope (Hitachi, Tokyo, Japan) was used for these observations. Samples were deposited on thin copper grids and examined at an accelerating voltage of 30 kV over a wide range of magnifications [36,37,38].

#### 2.3.2. Analysis of the Surface and Grain Size of the Material

Specific surface area (SSA) measurements were performed using the physical adsorption method with nitrogen gas at liquid nitrogen temperature (77 K) [39,40]. The specific surface area was determined based on the multipoint Brunauer–Emmett–Teller (BET) isotherm measured over the relative pressure (P/Po) range of 0.05 to 0.30 [41]. Prior to measurement, the samples underwent degassing under vacuum for 24 h at 150 °C to remove adsorbed contaminants [42]. Measurements were carried out using an ASAP 2010 apparatus (Micromeritics Inc., Norcross, GA, USA), which is designed for high-precision surface area and porosity analysis based on gas adsorption theory.

Grain size measurements of chitosan powder samples were performed using laser diffraction (LD). The samples were prepared as aqueous suspensions, dispersed with high-energy ultrasound, and the addition of a fluidizer was added to ensure homogeneous distribution. Measurements were carried out using a Mastersizer 2000 instrument equipped with a Hydro S attachment (Malvern Instruments, Malvern, UK), which enables precise particle size analysis over a wide range (from 0.01 µm to 2000 µm) by detecting scattered laser light [43,44,45].

#### 2.3.3. UV-Vis Analysis

The optical properties of the resulting colloidal AgNPs were characterized using UV–Vis spectrophotometry over a wavelength range of 300–800 nm (Shimadzu UV-2501, Kyoto, Japan) [46] and shown in Figure 2. For analysis, approximately 3 mL of the nanoparticle suspension was transferred into a quartz cuvette (path length 1 cm) and placed in a thermostated sample holder (20 °C)—Figure 2.

Obtained spectrum with a characteristic maximum wavelength at 430 nm was registered after 20 min, since colloidal silver was synthesized. The intensity of the peak was more intense compared to previous obtained results [35], as well as a redshift was observed, suggesting further particle growth or aggregation. Then, the obtained solution (4 mL) containing silver nanoparticles was mixed with 50 mg of commercial chitosan (C_c_), chitosan from *H. erinaceus* (C_He_), as well as chitosan from *P. ostreatus* (C_Po_).

Alongside the nanoparticle suspensions, the precursor solutions containing silver ions and ascorbic acid, as well as samples modified with chitosan, were analyzed [47]. Dimethyl sulfoxide (DMSO) was used as the blank reference for all measurements to ensure accuracy [48]. Spectral analyses employed a quartz cuvette with a 1 cm optical path length, housed in a thermostatically controlled cell to maintain constant temperature conditions [49]. Before nanoparticle characterization, each reagent and solution was individually examined to confirm optical purity, ensuring that measured absorbance was specific to nanoparticle formation [50].

#### 2.3.4. FTIR-ATR Analysis

Vibrational infrared spectra of chitosan samples were recorded between 400 and 4000 cm^−1^ at room temperature using an FTIR Tensor II spectrometer (Bruker, Billerica, MA, USA) equipped with Attenuated Total Reflectance (ATR) [51]. Powder samples were homogenized to a uniform grain size before measurement to ensure consistency and accuracy in spectral data [52]. The Bruker Tensor II is known for its high sensitivity and robustness, providing precise spectral resolution suitable for identifying functional groups and assessing chemical structure in biomaterials [53].

#### 2.3.5. AAS Analysis

The initial materials—commercial chitosan derived from crustaceans and chitosan extracted from the biomass of *in vitro* cultures of *H. erinaceus* and *P. ostreatus*—were mineralized in a closed, wet system using microwave-assisted digestion (Magnum II Mineralizer, ERTEC, Wrocław, Poland) [54,55]. For this purpose, homogenized biomaterial (0.5 g of each sample, including biomass and chitosan) was weighed into Teflon vessels. The mineralization process consisted of three stages (Stage I: 10 min, Stage II: 20 min, Stage III: 15 min) and was conducted at 280 °C [55]. The resulting colorless solutions were quantitatively transferred to quartz evaporators and evaporated on a hotplate at 120 °C to near dryness. The residue was then diluted in 10 mL volumetric flasks using quadruple-distilled water [56]. Samples were analyzed for bioelement content (Mg, Zn, Cu, Fe, Ca, Na, K, Mn) using flame atomic absorption spectrometry (F-AAS) with an iCE3500 spectrometer (Thermo Scientific, Gloucester, UK). Each sample was prepared in triplicate and measured three times (n = 9) to ensure reproducibility [56].

#### 2.3.6. DSC Analysis

Thermal behavior analysis of the synthesized materials was conducted using a Mettler Toledo DSC 3 differential scanning calorimeter (Greifensee, Switzerland). Each sample, approximately 3.5 mg in mass, was sealed in matching 40 μL aluminum pans, with an empty pan serving as the reference. The samples were positioned in the calorimeter, and data acquisition and analysis were managed via the STAR Thermal Analysis Software (Version 2025, Mettler Toledo, Columbus, OH, USA). The experimental protocol involved heating the unmodified chitosan samples from 20 °C to 300 °C, while silver-modified samples were heated from 0 °C to 250 °C under a nitrogen atmosphere to prevent oxidation. A constant heating rate of 10 °C/min was maintained throughout all experiments to ensure consistent thermal transitions and accurate measurements (Mettler Toledo) [57].

#### 2.3.7. Microbiological Analysis

The susceptibility of reference strains *Staphylococcus aureus* ATCC 29213 and *Escherichia coli* ATCC 25922 to silver nanoparticles (AgNPs) combined with chitosan was evaluated using the disk diffusion Method [58]. Mueller–Hinton agar plates (bioMérieux, Warsaw, Poland) were inoculated with standardized bacterial suspensions prepared in 0.85% sterile saline [58]. Sterile blank discs (Argenta, Poznań, Poland) containing AgNPs alone, chitosan alone, or their combination were placed on the agar surface. Crucially, discs impregnated with dimethyl sulfoxide (DMSO, Merck, Darmstadt, Germany)—the common solvent for nanoparticles and combinations—were included as controls to account for solvent effects on bacterial growth [58]. The plates were incubated at 37 °C for 24 h, after which the zones of inhibition (ZOI) around each disc were measured. Larger ZOI indicated stronger antimicrobial activity against the tested strains. The antibacterial effects of the AgNPs/chitosan composites were compared with those of individual agents and the inert DMSO control to assess any synergistic interactions. The DMSO control produced no significant inhibition, confirming that the observed antibacterial activity was due to active compounds [58]. For chitosan, ZOI diameters were interpreted based on the European Committee on Antimicrobial Susceptibility Testing (EUCAST) breakpoint criteria (ver. 15.0, effective from 1 January 2025) [58].

### 2.4. Reagents

#### 2.4.1. Liquid Culture Media

Zinc chloride (ZnCl_2_) and calcium chloride (CaCl_2_) salts were purchased from Sigma-Aldrich, Darmstadt, Germany (Merck KGaA, 2025, Darmstadt, Germany).

#### 2.4.2. Mineralization of Freeze-Dried Biomass

A mixture of concentrated nitric acid (65%, HNO_3_, Suprapure^®^, Merck, Darmstadt, Germany) and hydrogen peroxide (30%, H_2_O_2_, Suprapure^®^, Merck, Darmstadt, Germany) was used along with quadruple-distilled water characterized by conductivity below 1 μS/cm (HLP 5 distillation system, Hydrolab, Straszyn, Poland) [59].

#### 2.4.3. AAS Method Measurement

Standard solutions of metals: Ca(II), Zn(II) at a concentration of 1 g/L (Sigma-Aldrich, Darmstadt, Germany).

### 2.5. Statistical Analysis

The effects of chitosan type and the addition of silver nanoparticles (AgNPs) on the inhibition zone diameters for Staphylococcus aureus and Escherichia coli were analyzed using generalized linear modeling (GLM) with Statistica software version 13.3 (TIBCO Software Inc., Palo Alto, CA, USA) [60]. Preliminary data exploration indicated that the inhibition zone measurements did not follow a normal distribution, rendering traditional parametric methods unsuitable. To model the data appropriately, the Akaike Information Criterion (AIC) was used to compare candidate GLM models with various distributions and link functions. The optimal model, selected based on the lowest AIC, used a gamma distribution paired with a log link function [60]. This choice accommodates continuous, positive, and skewed data typical of inhibition zone diameters. The GLM incorporated two factors: the type of chitosan (C_Po_, C_c_, and C_He_) and the binary presence or absence of AgNPs, including their interaction effects on inhibition zones. Each treatment was replicated five times to ensure statistical robustness [60]. This approach provides a reliable method for analyzing antibacterial efficacy data that exhibit non-normal distributions, enabling more accurate inference about treatment effects.

## 3. Results

### 3.1. Characterization Materials

In this study, three materials were analyzed: commercial chitosan (Cc—derived from crustaceans, molecular weight, MwCc = 210 kDa—degree of deacetylation, DD_Cc_ = 83%), and chitosan from two mushrooms: (C_He_—derived from *H. erinaceus*, molecular weight, Mw_Cc_ = 180 kDa—degree of deacetylation, DD_CHe_ = 86%) and (C_Po_—derived from *P. ostreatus*, molecular weight, Mw_CPo_ = 3 kDa—degree of deacetylation, DD_CPo_ = 98%)

In the analyzed initial samples, the specific surface areas of chitosan powders were determined using the BET method: C_k_—1.1587 m^2^/g, C_He_—0.7315 m^2^/g, and C_Po_—0.3866 m^2^/g. The surface areas obtained using the Langmuir equation were 1.9348 m^2^/g, 0.9868 m^2^/g, and 0.4823 m^2^/g for C_k_, C_He_, and C_Po,_ respectively.

Table 1 summarizes the total pore volume (V_micro_), micropore area (S_micro_), and mean pore diameter (A_pore_) determined for the chitosan sample.

**Figure 3:**
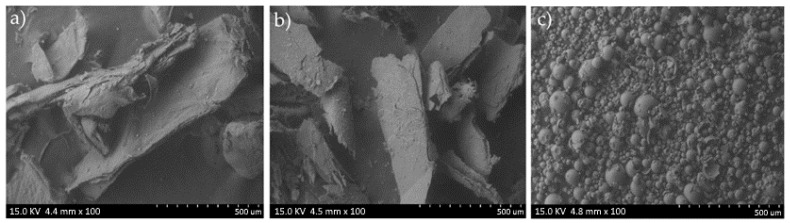
STEM analysis of the starting material: (**a**) Cc—chitosan from crustaceans, (**b**) C_He_—chitosan extracted from biomass from *in vitro* cultures of *H. erinaceus,* and (**c**) C_Po_—chitosan obtained from *in vitro* cultures of *P. ostreatus*.

The analyzed commercial chitosan (C_c_) is characterized by a very low micropore volume—its structure is practically nonporous—and a relatively small micropore surface area (Table 1), which indicates a limited availability of adsorption sites; therefore, its adsorption capacity may be restricted. A similar trend was observed for chitosan derived from *H. erinaceus* (C_He_), which exhibits very fine microporosity and a low specific surface area, also suggesting limited adsorption potential. In contrast, chitosan extracted from *P. ostreatus* (C_Po_) shows a considerably higher micropore volume (over 400 times greater than that of the other chitosans) and a very large pore diameter typical of macroporous materials. However, this sample displays a smaller micropore surface area (Table 1). Such a structure, characterized by significantly larger pores and higher total pore volume, may enhance its ability to adsorb larger molecules or ions despite the reduced micropore surface area. STEM images of the starting materials C_k_—commercial chitosan derived from crustaceans, C_He_—fungal chitosan extracted from biomass of *in vitro* cultures of *H. ericeus*, and C_Po_—fungal chitosan extracted from biomass of *in vitro* cultures of *P. ostreatus* are shown in Figure 3.

The analyzed scanning electron microscopy (STEM) images reveal distinct surface topographies among the studied chitosan samples, reflecting differences in their origin and processing methods. The STEM image of sample C_c_ (Figure 3a) shows the morphology of chitosan obtained by deacetylation of chitin sourced from crustaceans. The surface exhibits an irregular, flake-like structure with clearly visible folds and fibrous regions, typical of chitosan biopolymers subjected to drying. Numerous cracks and folds indicate the material’s brittle nature, suggesting mechanical stress during drying and sample preparation. Additionally, small, bright granules scattered on the surface likely correspond to residual mineral salts, such as calcium carbonate or sodium chloride, that were not fully removed during raw material purification. This morphology is consistent with known features of crustacean-derived chitosan, where drying induces structural irregularities and brittleness. The presence of residual minerals can influence the material’s physicochemical properties. Overall, STEM analysis provides valuable insight into how origin and processing affect chitosan’s surface characteristics, which in turn impact its functional properties.

The SEM image of sample C_He_, derived from chitosan extracted from *in vitro* cultured *H. erinaceus* biomass (Figure 3b), displays a layered, flake-like morphology. The sample forms large agglomerates several hundred micrometers in size, consisting of thin, exfoliated flakes. The surface is relatively rough, showing numerous cracks and micropores, which may suggest partial degradation of the polysaccharide structure during deacetylation. Plate-like fragments on the surface could result from delamination of the biopolymer during drying or remnants of the fungal cell wall structure. This morphology reflects the intrinsic structural characteristics of fungal-derived chitosan and its processing history.

The SEM image of sample C_Po_, derived from chitosan extracted from *in vitro* cultured *P. ostreatus* biomass (Figure 3c), displays numerous spherical particles with a wide size distribution, predominantly ranging from several tens to several hundreds of micrometers in diameter. Most particles have smooth surfaces, although some exhibit porosity or small openings, possibly indicating partial degradation or structural changes from the chitosan extraction process. Irregularly shaped fragments are also observed, which may represent remnants of cell walls or artifacts from sample preparation. The presence of hollow or partially open particles suggests the formation of chitosan microspheres, likely arising from coagulation or drying during extraction and purification.

### 3.2. Structure Analysis (FTIR-ATR)

Analysis of FTIR-ATR spectra of chitosan from mushrooms showed that they are similar to spectra from marine and other fungal sources [61,62,63]. Chitosan from mushrooms has a characteristic band in the region of 1615–1634 cm^−1^ which is linked to amine absorption in mushrooms chitosan-glucan complex, a band around 3351–3359 cm^−1^ representing OH wagging, and a CH stretching band in the 2871–2882 cm^−1^ region [63].

**Figure 4:**
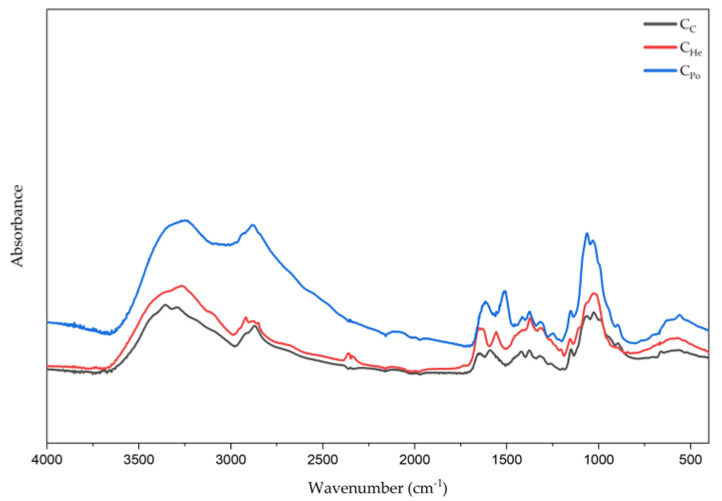
The FTIR-ATR spectra for the samples are presented as follows: C_c_—chitosan derived from crustaceans represented by the black line, C_He_—chitosan extracted from biomass of *in vitro* cultured *Hericium erinaceus*, shown as the red line, and C_Po_—chitosan obtained from *in vitro* cultured *Pleurotus ostreatus*, depicted by the blue line.

Physicochemical characterization of the chitosan samples was performed using FTIR-ATR spectroscopy to identify functional groups. The characteristic infrared peaks and corresponding functional groups are summarized in Table 2 and illustrated in Figure 4. The chitosan samples exhibited the O–H stretching band of alcohols at 3354 cm^−1^ (C_C_), 3351 cm^−1^ (C_Po_), and 3359 cm^−1^ (C_He_). The N–H stretching band of free amine groups appeared at 3291 cm^−1^ (C_C_), 3248 cm^−1^ (C_Po_), and 3110 cm^−1^ (C_He_). Bands observed at 2916 and 2871 cm^−1^, as well as 2927 and 2918 cm^−1^, correspond to asymmetric stretching of CH_3_ and CH_2_ groups. An intense peak at 1589 cm^−1^ (C_C_), 1515 cm^−1^ (C_He_), and 1557 cm^−1^ (C_Po_) corresponds to the NH_2_ bending vibration of the amine group, a distinctive feature of the chitosan biopolymer. Additionally, bands in the 1322–1417 cm^−1^ region are attributed to the bending vibrations of CH_3_ methyl groups. The C–O stretching band was identified near 1063–1064 cm^−1^, while the band at 1155 cm^−1^ in sample CD corresponds to asymmetric C–O–C stretching. Table 2 provides a detailed summary of these spectral features for all analyzed samples.

Infrared spectra are useful for comparing and identifying functional groups, as well as assessing the properties of chitosan derived from different mushroom species and various preparation methods. In the examined samples, shifts toward higher wavenumber values were observed, which may be directly related to the extraction method and the concentration of NaOH used during the process.

### 3.3. AAS Analysis

The analysis of the prepared samples using the AAS method revealed significant differences in metal content among the materials—commercial chitosan derived from crustaceans (C_c_) and chitosan extracted from the biomass of *in vitro* cultures of the fungi *H. erinaceus* (C_He_) and *P. ostreatus* (C_Po_)—as summarized in Table 3.

The content of analyzed metals in chitosan derived from crustacean shells is generally lower—except for sodium (Na), potassium (K), and manganese (Mn)—compared to chitosan extracted from fungal biomass cultivated *in vitro* (Table 3). The higher concentrations of other elements in fungal chitosan can be attributed to the composition of the culture medium, which contains these elements, particularly zinc (Zn) and calcium (Ca). Calcium was additionally supplemented to enrich the final material. This difference arises because crustacean-derived chitosan undergoes demineralization to remove abundant minerals like calcium carbonate, whereas fungal chitosan typically retains more bioelements due to cultivation conditions and processing methods.

### 3.4. Thermal Stability Analysis of Chitosan

**Figure 5:**
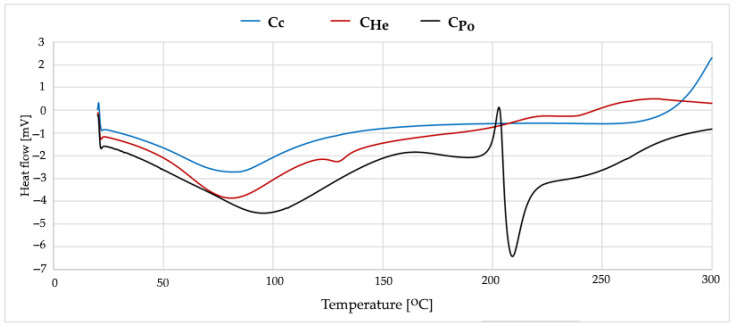
DSC curves from first heating measurements of C_c_—chitosan from crustaceans, C_He_—chitosan extracted from biomass from *in vitro* cultures of *H. erinaceus*, and C_Po_—chitosan obtained from *in vitro* cultures of *P. ostreatus*.

Commercial chitosan exhibits a typical thermal profile with a peak observed between 80 and 85 °C (Figure 5). This high crystallinity is attributed to the regular arrangement of polymer chains and the presence of strong intermolecular hydrogen bonds between –OH and –NH_2_ groups. The material has already undergone thermal treatment, as no significant thermal events occur above 150 °C, indicating its thermal stability within this temperature range.

Chitosans derived from fungi display comparable thermal transition temperatures, with the main endothermic peak appearing between 82 and 90 °C (Figure 5). The broader endothermic peak indicates a less homogeneous material structure, which is characteristic of fungal-derived chitosans. Key differences between commercial and fungal chitosans may be related to arise from the distinct structural organization within fungal cell walls and the possible presence of trace amounts of fungal proteins and lipids. Fungal chitosans typically exhibit lower crystallinity and weaker intermolecular hydrogen bonding compared to commercial chitosan.

### 3.5. Effect of Silver Modification

**Figure 6:**
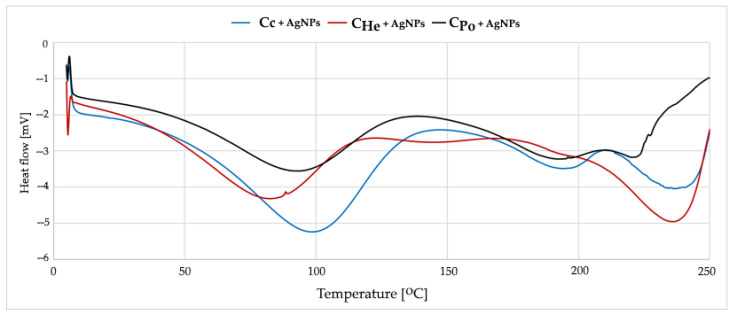
DSC curves from first heating measurements of C_c_—chitosan from crustaceans, C_He_—chitosan extracted from biomass from *in vitro* cultures of *H. erinaceus*, C_Po_—chitosan obtained from *in vitro* cultures of *P. ostreatus* enriched with AgNPs.

Commercial chitosan modified with silver nanoparticles (Chitosan + AgNPs) exhibits significant changes in its thermal profile (Figure 6). The main endothermic peak shifts to a higher temperature range of 115–120 °C, indicating a stiffening of the polymer structure. This change is attributed to the formation of coordination complexes between silver ions and the amino groups of chitosan. The incorporation of silver nanoparticles enhances the thermal stability of the material, likely due to strong interactions between silver and the polymer matrix that restrict polymer chain mobility.

Fungal chitosans modified with silver exhibit similar thermal changes, with the endothermic peak shifting to 95–100 °C (Figure 6). Silver modification slightly enhances thermal stability, increasing the onset temperature of degradation by approximately 5 °C. The main endothermic peak corresponds to the loss of water bound to hydroxyl and amino groups and to the reorganization of the crystalline structure. The broader peak observed in fungal chitosans indicates a less uniform energy distribution of hydrogen bonds. At higher temperatures, polymer backbone degradation occurs, involving deacetylation and depolymerization processes. Silver modification delays this degradation by stabilizing the polymer structure.

### 3.6. Microbiological Activity

**Figure 7:**
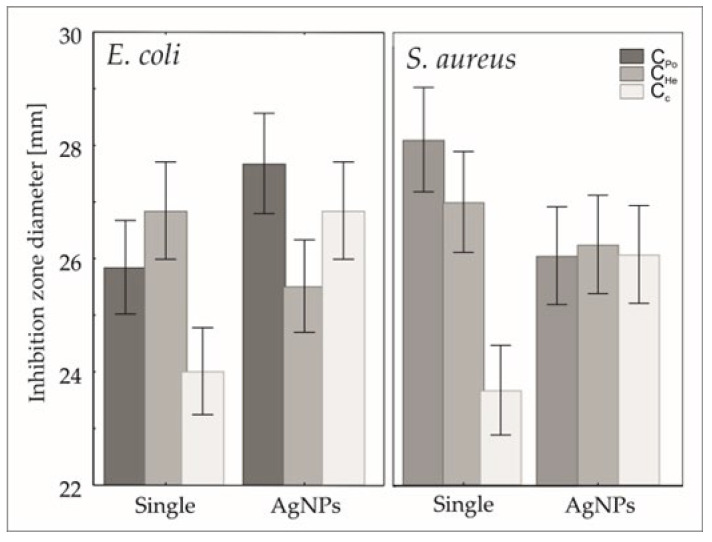
Predicted mean + confidence interval of inhibition zone distribution of *E coli* and *S. aureus* strains.

Generalized linear modeling revealed a significant effect of the chitosan type and the combined presence of silver nanoparticles (AgNPs) on the inhibition of pathogenic bacterial strains, including both Gram-negative *Escherichia coli* and Gram-positive *Staphylococcus aureus*, as summarized in Table 4. Specifically, for *E. coli*, the incorporation of AgNPs enhanced the inhibition zones when combined with chitosan types C_Po_ and Cc. Conversely, the addition of AgNPs to chitosan type C_He_ resulted in a significant reduction of antimicrobial efficacy (Figure 7). In the case of *S. aureus*, an increase in inhibition was observed exclusively with the combination of AgNPs and chitosan type C_c_. Both fungal-derived chitosan samples, C_Po_ and C_He_, exhibited reduced antibacterial activity upon integration of AgNPs, as demonstrated in Figure 7.

## 4. Discussion

The morphology of chitosan derived from the biomass of *in vitro* cultures of *H. erinaceus* and *P. ostreatus* differs significantly from that of chitosan obtained from traditional sources such as crustaceans (Figure 3a). Scanning electron microscopy (STEM) images of the fungal-derived chitosan (Figure 3b,c) reveal a flake-like, irregular structure characterized by large agglomerates and numerous surface cracks. This morphology indicates a less ordered macromolecular arrangement and higher porosity, which are characteristic features of fungal-based chitosan [64]. Such structural differences are consistent with the amorphous nature of fungal chitosan observed in previous studies, where high porosity and irregular surface features contrast with the more compact and ordered structure of crustacean-derived chitosan [64]. These features may influence the physicochemical properties and functional applications of fungal chitosan, including enhanced bioactivity and higher surface area for interactions [53].

In contrast, chitosan derived from crustacean exoskeletons typically exhibits a more fibrous and uniformly distributed structure with a distinct orientation of microfibers. This morphology results from the highly organized chitin matrix in the exoskeleton. In this matrix, chitin fibers are layered and tightly bound to structural proteins and minerals, primarily calcium carbonate (CaCO_3_) [65,66]. The demineralization and deproteinization processes used during chitosan extraction from crustaceans produce smoother surfaces and a more homogeneous texture. These changes reflect the removal of minerals and proteins that mask the underlying ordered fibrous structure [67,68]. This structural arrangement contributes to the mechanical strength and rigidity characteristic of crustacean-derived chitosan compared to fungal chitosan, which is more porous and less ordered [53,69].

Fungal chitosan obtained from the cell walls of basidiomycetes exhibits a more amorphous and less consolidated structure compared to chitosan derived from crustaceans. This difference arises from the absence of a mineral phase and the presence of a distinct polysaccharide matrix that includes β-glucans and mannoproteins [53,64]. This morphological distinction is associated with enhanced sorption capacity and improved biocompatibility, although fungal chitosan generally has lower mechanical strength than crustacean-derived chitosan. Consequently, fungal chitosan exhibits improved physicochemical properties and holds significant potential for biomedical applications such as tissue engineering, drug delivery, and wound dressings [70,71,72].

The present results show that chitosan isolated from biomass of *in vitro* cultures of *H. erinaceus* and *P. ostreatus* contains higher amounts of bioelements like zinc and calcium. These amounts exceed those found in chitosan from crustaceans or from unsupplemented *in vitro* cultures. This aligns with previous findings demonstrating that supplementation of culture media with selected micro- and macroelements significantly affects the chemical composition of secondary metabolites and structural compounds. The incorporation of zinc and calcium into the chitosan matrix may have practical implications for biomedical use. Zinc plays a crucial role in tissue regeneration, collagen synthesis, and modulation of inflammatory responses [73]. Calcium regulates hemostasis and cell proliferation, which are essential processes in wound healing. Thus, their presence could enhance the bioactivity and efficacy of chitosan in bioactive wound dressings. Elevated zinc content may also boost chitosan’s antibacterial properties, vital for infection prevention. In this study, the material exhibited significantly stronger antibacterial activity, even surpassing that of chitosan combined with silver nanoparticles [74].

These enhanced properties likely result from both the bioelements and the presence of free oxygen-containing groups. FTIR-ATR analysis revealed asymmetric stretching vibrations attributable to oxygen functional groups, which contribute to the material’s bioactivity. The bactericidal activity of mushroom-derived chitosan is multifactorial. It involves reactive oxygen species (ROS) such as hydrogen peroxide (H_2_O_2_), hydroxyl radicals (-OH), and superoxide anion radicals (O_2_^−^ −) generated by free oxygen moieties in the structure. These ROS directly damage bacterial cellular components—membranes, proteins, and nucleic acids—leading to cell death [75]. Moreover, ROS increase oxidative stress and disrupt microbial metabolism and repair mechanisms. They also promote lipid peroxidation of membranes, which enhances bactericidal effects. The cationic nature of chitosan induces protein denaturation and disrupts membrane permeability. These effects are exacerbated by ROS, making chitosan’s antibacterial action both multifaceted and effective [75].

The results of this study demonstrate that the antimicrobial efficacy of chitosan is significantly modulated by the type of chitosan and its combination with silver nanoparticles (AgNPs), leading to strain-specific effects against pathogenic bacteria such as *E. coli* and *S. aureus* [76]. The synergistic enhancement of inhibition zones observed for *E. coli* when AgNPs were combined with chitosan types C_Po_ and C_c_ aligns with recent findings that chitosan facilitates the adsorption and localized release of silver ions, destabilizing bacterial membranes and amplifying silver’s bactericidal action [77]. This synergy likely arises from electrostatic interactions between the positively charged chitosan-AgNPs complexes and the negatively charged bacterial cell surfaces, which promote enhanced antimicrobial activity [78]. The finding that AgNPs reduced antimicrobial activity when integrated with the fungal-derived chitosan type C_He_ suggests that structural or physicochemical properties inherent to this chitosan variant may interfere with silver ion release or bacterial interaction. Similar differential antimicrobial responses have been attributed to variations in chitosan molecular weight, degree of acetylation, and functional group availability, which affect nanoparticle stability and ion release kinetics [79]. The exclusive increase in *S. aureus* inhibition with AgNPs combined with chitosan type Cc further highlights the strain-specific mechanisms governing antimicrobial efficacy, potentially related to the distinct cell wall architectures of Gram-positive versus Gram-negative bacteria affecting nanoparticle interaction [80].

The reduced antibacterial activity of fungal-derived chitosans C_Po_ and C_He_ upon AgNPs addition may reflect differences in nanoparticle–chitosan matrix interactions that influence nanoparticle aggregation or bioavailability, ultimately impacting antimicrobial potency [81]. Overall, these results underscore the importance of carefully selecting chitosan types in designing antimicrobial nanocomposites, as their intrinsic properties critically determine the synergistic potential with silver nanoparticles against various pathogens. This study contributes to understanding how chitosan’s molecular characteristics and source influence the antimicrobial performance of chitosan-AgNPs composites, providing guidance for optimizing formulations targeting clinically relevant bacterial strains [76,81]. Despite the promising results, further *in vitro* and *in vivo* studies are essential to comprehensively evaluate the biocompatibility and therapeutic efficacy of the developed material. Particularly critical is the determination of the release profile of Zn^2+^ and Ca^2+^ ions from the chitosan matrix and their effects on skin cells and wound microbiota [82,83]. Chitosan derived from fungal biomass cultivated *in vitro* represents a promising alternative to crustacean-derived chitosan [84]. Supplementing the culture medium with bioelements proves to be an effective approach to modulate the composition and functionality of chitosan, especially when combined with optimized extraction methods [28]. current data suggest that such material could serve as a valuable platform for developing modern, bioactive wound dressings designed to support and accelerate wound healing [85]. Chitosan’s inherent biocompatibility, biodegradability, and antimicrobial properties, combined with controlled release of bioactive ions, make it particularly suitable for applications in tissue engineering and wound care. Moreover, recent studies highlight that fungal chitosan-based nanomaterials enhance cellular proliferation, angiogenesis, and collagen synthesis, further supporting its therapeutic potential [82,86].

## 5. Conclusions

Chitosan extracted from *in vitro* cultures of *Pleurotus ostreatus* and *Hericium erinaceus* provides a viable and consistent alternative to traditional crustacean-derived chitosan, addressing variability and quality concerns associated with marine sources.

Fungal-derived chitosans, particularly from *P. ostreatus*, exhibit distinctive morphological features and enriched bioelement content, which enhance their antimicrobial efficacy.

Combining chitosan with silver nanoparticles (AgNPs) significantly enhances antibacterial activity against *Escherichia coli* and *Staphylococcus aureus* in both commercial and *P. ostreatus* chitosans.

The impact of AgNPs on antimicrobial activity varies depending on the chitosan source, highlighting the importance of selecting the appropriate matrix for specific applications.

Incorporation of silver nanoparticles enhances the thermal stability of chitosan materials, supporting their suitability for biomedical applications.

Fungal chitosan-AgNPs composites hold promise as advanced wound dressing materials with improved microbial control and wound healing properties.

Future research should aim to optimize synthesis parameters, assess *in vivo* cytocompatibility, and elucidate the mechanisms underlying interactions between chitosan matrices and nanoparticles to fully harness their therapeutic potential.

## Figures and Tables

**Table 1:** Characteristic Parameters of biomaterials.

Type of Chitosans/Parameters	C_c_	C_He_	C_Po_
V_micro_, cm^3^/g	0.000014	0.000015	0.006631
S_micro_, m^2^/g	1.0957	0.9842	0.3273
A_pore_, nm	1.5486	0.5617	81.0301

Shortcuts: Cc—commercial chitosan, C_He_—chitosan from *H. erinaceus*, C_Po_—Chitosan from *P. ostreatus*, V_micro_—pore volume, A_pore_—average pore diameter, S_micro_—micropore area.

**Table 2:** The FTIR-ATR analyses of the functional groups of the analyzed chitosans.

Functional Group/Type of Vibration	C_c_ [cm^−1^]	C_He_ [cm^−1^]	C_Po_ [cm^−1^]
C-H (bending)	894	900	896
C-O (stretching)	1027	1026	1032
C-O (stretching)	1063	-	1064
C-O-C (stretching asymmetric)	1150	1155	1152
C-O (stretching)	1260	1265	1249
C=N (stretching)	1322	1316	1318
CH_3_ (symmetrical deformation)	1377	1374	1374
CH_2_ (deformation)	1422	1417	1417
NH (bending)	1589	1557	1515
C=O (stretching)	1651	1652	1615
CH (stretching asymmetric)	2871	2851	2882
CH (stretching symmetric)	2916	2918	2927
NH (stretching)	3291	3272	3248
OH (stretching)	3354	3359	3351

Shortcuts: Cc—commercial chitosan, C_He_—chitosan from *H. erinaceus*, C_Po_—Chitosan from *P. ostreatus.*

**Table 3:** Metal content analysis of chitosan samples derived from mushroom biomass and crustacean shells, expressed in mg per 100 g of dry weight.

Functional Group/Type of Vibration	C_c_	C_He_	C_Po_
Mg	1.12 ± 0.10	1.64 ± 0.10	1.96 ± 0.10
Zn	2.96 ± 0.10	6.45 ± 0.50	8.36 ± 0.10
Cu	0.44 ± 0.04	0.85 ± 0.10	1.02 ± 0.10
Fe	5.11 ± 0.20	9.23 ± 0.20	8.60 ± 0.30
Ca	0.90 ± 0.10	3.24 ± 0.10	5.30 ± 0.20
Na	1.10 ± 0.10	0.79 ± 0.05	1.36 ± 0.20
K	3.11 ± 0.20	1.43 ± 0.20	1.78 ± 0.10
Mn	1.42 ± 0.10	0.77 ± 0.05	0.95 ± 0.10

Shortcuts: Cc—commercial chitosan, C_He_—chitosan from *H. erinaceus*, C_Po_—Chitosan from *P. ostreatus.*

**Table 4:** Generalized linear mixed model summary of chitosan type and AgNPs addition to the inhibition diameter of *E. coli* and *S. aureus* strains.

Effect	d.f.	Wald Stat.	*p*
*E. coli*			
Intercept	1	227,880.1	0.000
Chitosan type	2	27.1	0.000
AgNPs	1	7.2	0.007
Chitosan type * AgNPs	2	10.4	0.005
*S. aureus*			
Intercept	1	244,102.3	0.000
Chitosan type	2	104.8	0.000
AgNPs	1	1.6	0.211
Chitosan type * AgNPs	2	70.9	0.000

Abbreviations: df—degrees of freedom, Walds Stat.—Wald coefficient of statistics, * indicates interaction between factors.

## Data Availability

The original contributions presented in this study are included in the article. Further inquiries can be directed to the corresponding authors.
